# Effects of dialysate to serum sodium (Na^+^) alignment in chronic hemodialysis (HD) patients: retrospective cohort study from a quality improvement project

**DOI:** 10.1186/s12882-018-0870-0

**Published:** 2018-04-02

**Authors:** Jochen G. Raimann, Linda H. Ficociello, Len A. Usvyat, Hanjie Zhang, Lisa Pacelli, Sandi Moore, Penny Sheppard, Qingqing Xiao, Yuedong Wang, Claudy Mullon, Paul Balter, Terry Sullivan, Peter Kotanko

**Affiliations:** 1 Renal Research Institute, 315 East 62nd Street,4th Floor, New York, NY 10065 USA; 2 Fresenius Medical Care North America, Waltham, MA USA; 3 University of California Santa Barbara, Santa Barbara, CA USA

**Keywords:** Sodium mass balance, Interdialytic weight gain, Blood pressure, Dialysate to serum sodium alignment

## Abstract

**Background:**

Evidence indicates favorable effects of dialysate (DNa^+^) to serum sodium concentration (SNa^+^) alignment, however, results from larger sample populations are needed. For this reason, we conducted a retrospective propensity score-matched cohort study from a quality improvement project to investigate the effects of alignment on population of maintenance hemodialysis patients.

**Methods:**

At 4 participating hemodialysis (HD) clinics, patients with SNa^+^ lower than the standard DNa^+^ of 137 mEq/L who received HD with DNa^+^ aligned to the average of the last 4 SNa^+^ measurements were evaluated (clinicaltrials.gov #NCT01825590). In this retrospective data analysis, an intention-to-treat (primary) and an as-treated “intervention” (secondary) cohort were created. “Aligned” patients from both cohorts (*N* = 163 for the primary and *N* = 137 for the secondary) were then propensity-score matched in a 1:1 fashion to “unaligned” patients from the Renal Research Institute database. The propensity score was generated based on age, gender, white race, Hispanic ethnicity, absence or presence of diabetes, hemodialysis vintage, interdialytic weight gain (IDWG; as a percentage of postdialysis body weight), catheter as primary dialysis access, predialysis systolic blood pressure, serum sodium concentration, hospitalization count during baseline. T-Test was employed for group comparisons of changes to the primary (volume-related and hemodynamic parameters) and tertiary outcomes. All-cause and fluid overload-related hospitalization admission rates were compared using Wilcoxon Rank Sum test and Cox regression analysis for repeated events.

**Results:**

In the primary analysis, aligned and unaligned subjects showed comparable demographics at baseline. Treatment effects were significant for IDWG [−0.12 (95% CI −0.24 to 0) L] and showed decreasing non-significant trends for pre-dialysis hemodynamic parameters. Count comparison and Cox regression analysis showed no clear advantage of alignment in terms of all-cause and fluid overload-related hospitalization.

**Conclusions:**

Results from the largest sodium alignment program to date suggest positive treatment effects on volume-related and hemodynamic parameters, but no clear effect on risk of hospitalization. Well-matched control patients minimized confounding effects. Small effects and lack of significant differences may be explained by a low baseline DNa^+^ limiting the interventional change.

**Electronic supplementary material:**

The online version of this article (10.1186/s12882-018-0870-0) contains supplementary material, which is available to authorized users.

## Background

During the interdialytic period sodium mass balance in hemodialysis (HD) patients is positive due to dietary intake and the inability to excrete sodium and water via the kidney. Convective removal of fluid and salt during HD treatments, considered isotonic and isonatremic, are employed to reduce fluid overload in this population. However, not only diet and convective removal affects sodium balance: Diffusive transfer, as found in the presence of a gradient between the dialysate and the plasma water concentration (GNa^+^) has also been reported to affect serum sodium concentrations [SNa^+^; [1, 2]] and subsequently serum osmolality [3]. While effective osmolality always decreases during HD due to the removal of various retention solutes [4], there are substantial variations between the decreases in those receiving HD with a positive, neutral and negative GNa^+^ [3]. These differences on the effects of the DNa^+^ on SNa^+^ (and osmolality) have been reported to trigger thirst and result in increased water intake [1, 2, 5, 6], in concert taking notable effect on the cardiovascular system [7]. Relationships between DNa^+^ and mean arterial pressure (MAP), flow mediated vasodilatation [8], blood pressure (BP) control [9–11], inflammation (as quantified by IL-6 and TNF-α) and the occurrence of intradialytic morbid events [9] have been reported. At variance to reported improvements on the risk of hospitalization conferred by lowering DNa^+^ in a nationwide quality improvement project at Fresenius Medical Care [12], and results from previously published literature, a recent analysis by the Dialysis Outcomes and Practice Patterns Study (DOPPS) reported a reduced risk for all-cause and fluid-overload related hospitalizations (using adjusted Cox regression models with time to first hospitalization as the outcome) with the use of high DNa^+^ in those with low SNa^+^ [5].

Independent of the prescribed DNa^+^, the SNa^+^ prior to the HD treatment is, besides to seasonal variation [13], a relatively constant [14] and a genetically determined sodium (or essentially osmolality) “setpoint” has been proposed for dialysis patients [2]. Given the operational roadblocks of measuring serum sodium before every HD treatment we have proposed an approach to estimate the SNa^+^ based on routine SNa^+^ during the preceding 4 months, thereby facilitating the operational implementation of DNa^+^ to SNa^+^ alignment. Implementation of the our approach with the aim to reduce intradialytic sodium loading in the operational structure of four dialysis clinics as a quality improvement project [11].

This retrospective, propensity score-matched, double cohort study based on data from a quality improvement project, analyzes the effects of dialysate sodium alignment in a population of maintenance HD patients receiving thrice-weekly treatment in 4 different clinics of the Renal Research Institute on interdialytic weight gain (IDWG) and blood pressure (BP) measurements as the co-primary outcomes. Secondary outcomes studied were the effects of GNa^+^ reduction on the risk of repeated all-cause and fluid-overload related hospitalization. Intradialytic saline administration and inflammatory markers were tertiary outcomes.

## Methods

### Study design, setting and participants

This retrospective, propensity score-matched, cohort study of a quality improvement project, analyzes the effects of DNa^+^ to SNa^+^ alignment in a population of maintenance HD patients receiving thrice-weekly treatment in 4 different clinics of the Renal Research Institute (clinicaltrials.gov #NCT01825590). In these clinics a previously proposed operational algorithm of DNa^+^ to SNa^+^ alignment [11] based on an estimated SNa^+^ (SNa^+^_estimated_) calculated as the average of the preceding 4 monthly SNa^+^ measurements, was implemented in the clinical routine. The standard DNa^+^ in the clinics was 137 mEq/L and all patients with a SNa^+^_estimated_ less than 137 mEq/L were subject to alignment and received an aligned, lower DNa^+^. No other changes to HD prescriptions in terms of treatment time, target eKt/V, saline administration and other prescriptions were made. Prescription of the post HD target weight prescription was at the treating physician’s discretion.

Although the study was retrospective and observational in nature a decision was made, based on suggestions found in the literature [15], to analyze the data as if patients were randomized to either receive alignment or not. This created a virtual “control” cohort using propensity score matching. Comparable to a randomized clinical study an intention-to-treat (ITT) analysis “enrolled” every “subject” with a low serum sodium once the dialysis clinic became part of the quality improvement project. Then an as-treated (AT) analysis was completed, which included only patients whose dialysate prescription was adjusted to their serum sodium. The ITT was considered the primary analysis while the AT served as the secondary analysis.

### Matching

All patients receiving alignment were matched with patients concurrently receiving HD in the 21 other clinics of the Renal Research Institute. A propensity score based on age, hemodialysis vintage (log-transformed due to skewness), gender, Catheter as primary dialysis access (yes/no), race (white/Non-White), diabetes (diabetic/non-diabetic), ethnicity (Hispanic/non-Hispanic), IDWG (% of post HD body weight), pre HD SBP, serum sodium and hospitalizations (dichotomized as either none or at least 1 hospitalization), during the 4 months baseline period was computed.

### Outcomes

The success of the “intervention” was quantified as the treatment effect on the dialysate to serum sodium gradient, which was calculated as prescribed dialysate sodium concentration minus predialysis serum sodium concentration. The co-primary outcomes of this propensity score-matched cohort study were volume related (with IDWG as the primary measure) and hemodynamic parameters (with pre HD SBP as the primary measure). Secondary outcomes were risk of (repeated) hospitalization due to all causes and fluid overload, respectively. Tertiary outcomes were the effects on intradialytic saline administration (as a surrogate of intradialytic morbid events), given the concerns of clinicians that a lower DNa^+^ may result in an increased occurrence of intradialytic hypotension, and neutrophil lymphocyte ratio, based on observations by Beduschi and colleagues, who reported an increase in inflammatory markers in those subject to sodium loading [16].

### Measurements

All measurements were taken from routine laboratory assessment and were performed in a centralized laboratory (Spectra Laboratories, Rockleigh, NJ, USA) by trained and certified personnel. SNa^+^ was measured by indirect potentiometry (40-fold dilution prior to SNa^+^ measurement), with a precision ranging between 1 and 2% coefficient of variation. Ethnicity was evaluated from medical records entries which are based on patients’ self-report.

Changes from baseline to the end of the observation period were assessed for volume-related parameters (interdialytic weight gain, ultrafiltration volume), hemodynamic parameters (pre- and post HD systolic, diastolic and mean arterial pressure; pre and post HD pulse pressure and intradialytic change of systolic and diastolic blood pressure) were captured. In addition, pre and post HD body weight before and after HD, Saline administered for the occurrence of intradialytic morbid events, the neutrophil lymphocyte ratio and hemoglobin were extracted from the medical records. All-cause and fluid-overload related hospitalizations were classified as per the ICD-9 codes.

### Subset analyses

In addition to the ITT and AT analyses, subset analyses were conducted to reduce the probability of confounding by factors that had not been subject to controlled by matching. These subset analyses included treatment effect evaluation in a) in patients with a GNa^+^ greater than 1 mEq/L (the intervention will be more effective in those with a larger GNa^+^), b) those with pre HD SBP greater than 150 mmHg (based on the threshold chosen by dePaula and colleagues [9]).

### Statistical methods

For the formal comparison of changes and the evaluation of the treatment effect (Change from Baseline to end of the intervention period in unaligned patients minus the change in aligned patients) Student’s T-Test was employed. Using medical records, hospitalizations were compared using a generalized linear model. Changes from baseline to study period, between cohorts at baseline and during follow-up were analyzed. The risk of recurrent hospitalization was analyzed using the Andersen-Gill method. All data are reported as mean ± SD or mean (95% CI) as appropriate. Analyses were conducted with R version 3.4.1 [codename “Single Candle”; R Foundation for Statistical Computing; Vienna, Austria [17]] additionally employing the packages *plyr* and *survival*.

## Results

### Study population

The quality improvement project was carried out with 215 patients receiving alignment between 04/2010 to 04/2012. From these, 163 patients were successfully matched with a group of unaligned patients from the Renal Research Institute database (*N* = 8848) with an equivalent DNa^+^ prescription (*N* = 4210) and were analyzed in the primary analysis. See Additional file 1 Figure S1 and Figure S2 for Quantile-Quantile plots of the cohorts before and after matching. The AT cohort was comprised of 137 patients which also matched with “unaligned” patients from the database. Treatment times were not altered from baseline to follow-up (aligned 210 ± 31 versus 216 ± 31 for the unaligned cohort). Except for a difference in hemodialysis vintage between both cohorts, the subjects’ demographic characteristics were well-balanced without substantial differences (Table 1 and Table 2).

**Table 1:** Demographics of patients included in this retrospective analysis of this quality initiative (intention-to-treat cohort) and the propensity score matched control cohort

Intention-To-Treat Cohort	Aligned (163 patients)	Not-Aligned (163 patients)	*P* (aligned vs. not aligned)
Age [years]	66.41+/−15.29	65.32+/−13.37	0.49
Hemodialysis vintage [years]*	2.8 [1.1 to 5.1]	1.6 [0.4 to 4.4]	< 0.001
Male gender [yes/no]	52.15%	54.6%	0.74
Catheter [yes/no]	22.09%	25.15%	0.6
White Race [yes/no]	60.74%	60.12%	1
Diabetes [yes/no]	65.64%	66.26%	1
Hispanic Ethnicity [yes/no]	8.59%	7.98%	1
Interdialytic weight gain (% of post-dialysis body weight)	2.53+/−0.93	2.67+/−0.97	0.19
Pre-dialysis systolic blood pressure [mmHg]	145.17+/−20.05	145.12+/−19.33	0.98
Serum sodium [mEq/L]	135.8+/−1.35	135.76+/−2.47	0.86
Patients with at least 1 hospitalization [yes/no]	14.11%	20.25%	0.19

*Data presented as median [25th to 75th percentile]; group comparison by non-parametric testing

**Table 2:** Demographics of patients included in this retrospective analysis of this quality initiative (as-treated cohort) and the propensity score matched control cohort

As-Treated Cohort	Aligned (137 patients)	Not-Aligned (137 patients)	*P* (aligned vs. not aligned)
Age [years]	68.41+/−14.53	66.59+/−14.74	0.31
Hemodialysis vintage [years]*	3.2 [1.4 to 5.6]	3.0 [1.2 to 6.6]	0.66
Male gender [yes/no]	51.82%	54.01%	0.81
Catheter [yes/no]	19.71%	16.79%	0.64
White Race [yes/no]	60.58%	56.93%	0.62
Diabetes [yes/no]	63.5%	64.96%	0.9
Hispanic Ethnicity [yes/no]	6.57%	3.65%	0.41
Interdialytic weight gain (% of post-dialysis body weight)	2.52+/−0.86	2.64+/− 0.92	0.26
Pre-dialysis systolic blood pressure [mmHg]	143.42+/−21.27	146.94+/−20.88	0.17
Serum Sodium [mEq/L]	135.65+/−1.6	135.4+/−2.94	0.38
Patients with at least 1 hospitalization [yes/no]	31.39%	35.77%	0.52

*Data presented as median [25th to 75th percentile]; group comparison by non-parametric testing

### Change in dialysate sodium prescription (intervention)

**Fig. 1:**
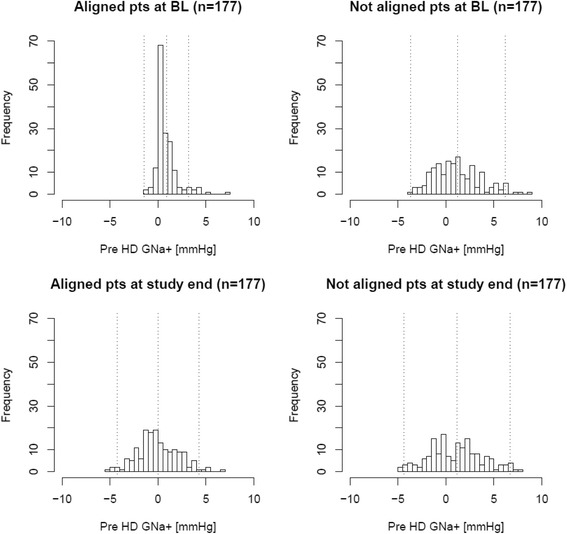
Distribution of pre hemodialysis dialysate to serum sodium gradient (pre HD GNa^+^) before and after prior to intervention start and in the control group

The change in dialysate sodium prescription was successfully implemented following a published algorithm [11] in all 4 clinics. While alignment had a reducing treatment effect on SNa^+^ in ITT and AT, the effect on GNa^+^ was significant in the AT cohort only (Table 3 and Table 4b**;** Fig. 1).

**Table 3:** Treatment effect of dialysate to serum sodium alignment on sodium-related parameters based on the data of those patients part to this quality initiative (intention-to-treat cohort; *N* = 163) and the propensity score matched control cohort (*N* = 163)

Parameter	Study Regimen	Baseline	Follow-up	Difference (95% CI)	Treatment effect (95% CI)
SNa^+^ [mEq/L]	aligned	135.80+/−1.35	135.77+/−2.57	−0.03 (−0.48 to 0.42)	−0.12 (−0.60 to 0.36)
	unaligned	135.76+/−2.47	135.80+/−2.8	0.09 (−0.48 to 0.66)	
GNa^+^ [mEq/L]	aligned	0.93+/−1.21	−0.03+/−2.20	−0.95 (−1.33 to −0.58)	−0.43 (−0.89 to 0.03)
	unaligned	1.35+/−2.62	0.82+/−2.86	−0.52 (−1.1 to 0.05)

**Table 4:** Treatment effect of dialysate to serum sodium alignment on sodium-related parameters based on the data of those patients part to this quality initiative (as-treated cohort; *N* = 137) and the propensity score matched control cohort (*N* = 137)

Parameter	Study Regimen	Baseline	Follow-up	Difference (95% CI)	Treatment effect (95% CI)
SNa^+^ [mEq/L]	aligned	135.65+/−1.60	135.68+/−2.49	0.03 (−0.47 to 0.53)	−0.28 (−0.8 to 0.24)
	unaligned	135.40+/−2.94	135.70+/−2.90	0.31 (−0.39 to 1.01)	
GNa^+^ [mEq/L]	aligned	1.35+/−1.60	−0.41+/−2.11	−1.76 (−2.21 to −1.31)	−1.45 (−1.98 to −0.91)
	unaligned	1.6+/−2.94	1.29+/−2.94	−0.31 (−1.01 to 0.39)

### Primary outcomes

**Fig. 2:**
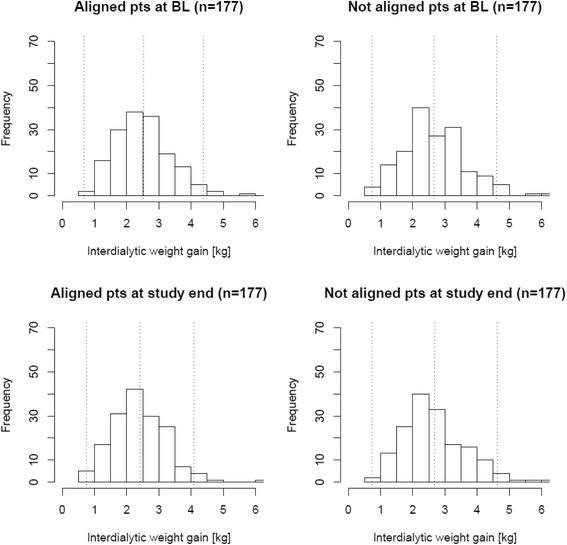
Distribution of interdialytic weight gain (IDWG) before and after prior to intervention start and in the control group

**Fig. 3:**
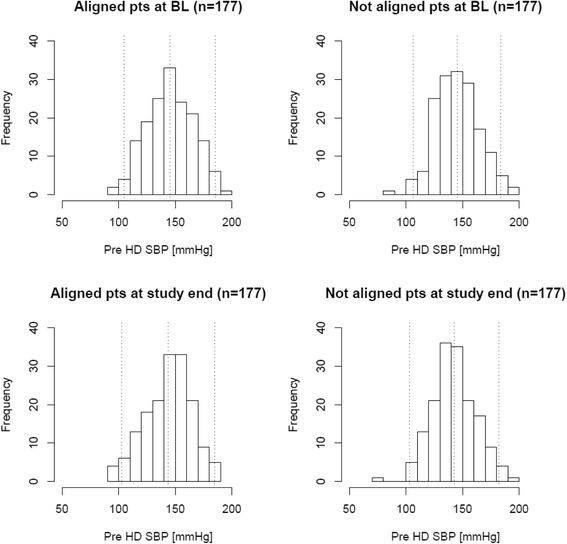
Distribution of pre hemodialysis systolic blood pressure (pre HD SBP) before and after prior to intervention start and in the control group

Dialysate to serum sodium alignment had a reducing effect on IDWG from BL to follow-up in the ITT analysis [− 0.12 (− 0.24 to 0); Table 5 and Fig. 2] and in the AT analysis [− 0.18 (− 0.30 to − 0.05]. This dynamic was consistent with a reduction of UFV in both primary (Table 5**)** and secondary analysis (Table 6). There were no significant treatment effects on pre and post HD body weights (Table 9 and Table 6) and no effects on pre and post HD blood pressure readings (Table 7 and Table 8**;** Fig. 3).

**Table 5:** Treatment effect of dialysate to serum sodium alignment on volume-related and hemodynamic parameters in those patients part to this quality initiative (intention-to-treat cohort; *N* = 163) and the propensity score matched control cohort (*N* = 163)

Parameter	Study Regimen	Baseline	Follow-up	Difference (95% CI)	Treatment effect (95% CI)
IDWG [L]	aligned	2.53+/−0.93	2.42+/−0.83	−0.11 (−0.3 to 0.08)	−0.12 (−0.24 to 0)
	unaligned	2.67+/−0.97	2.7+/−1	0.01 (−0.2 to 0.22)	
UFV [L]	aligned	2.44+/−0.86	2.32+/−0.76	−0.12 (−0.3 to 0.06)	−0.15 (−0.25 to −0.04)
	unaligned	2.58+/−0.96	2.6+/−0.9	0.03 (−0.18 to 0.23)	
Pre HD weight [kg]	aligned	81.82+/−22.63	81.21+/−22.13	−0.61 (−5.48 to 4.27)	−0.34 (−1.08 to 0.39)
	unaligned	80+/−21.44	79.7+/−20.9	−0.26 (−4.88 to 4.36)	
Post HD weight [kg]	aligned	79.41+/−22.18	78.81+/−21.47	−0.6 (−5.35 to 4.16)	−0.29 (−1.01 to 0.43)
	unaligned	77.44+/−20.96	77.1+/−20.4	−0.31 (−4.82 to 4.2)

**Table 6:** Treatment effect of dialysate to serum sodium alignment on volume-related and hemodynamic parameters in those patients part to this quality initiative (as-treated cohort; *N* = 137) and the propensity score matched control cohort (*N* = 137)

Parameter	Study Regimen	Baseline	Follow-up	Difference (95% CI)	Treatment effect (95% CI)
IDWG [L]	aligned	2.52+/−0.86	2.33+/−0.76	−0.19 (−0.38 to 0)	−0.18 (−0.3 to −0.05)
	unaligned	2.64+/−0.92	2.60+/−1.0	−0.01 (−0.24 to 0.21)	
UFV [L]	aligned	2.45+/−0.87	2.28+/−0.76	−0.17 (−0.37 to 0.02)	−0.15 (−0.26 to −0.04)
	unaligned	2.58+/−0.93	2.6+/−0.9	−0.02 (−0.24 to 0.2)	
Pre HD weight [kg]	aligned	81.29+/−20.67	80.48+/−20.17	−0.8 (−5.66 to 4.06)	−0.39 (−1.13 to 0.35)
	unaligned	75.36+/−19.3	75.0+/−18.70	−0.41 (−4.93 to 4.11)	
Post HD weight [kg]	aligned	78.84+/−20.16	78.22+/−19.80	−0.61 (−5.36 to 4.14)	−0.2 (−0.92 to 0.53)
	unaligned	72.82+/−18.9	72.4+/−18.30	−0.42 (−4.84 to 4.0)

**Table 7:** Treatment effect of dialysate to serum sodium alignment on volume-related and hemodynamic parameters in those patients part to this quality initiative (intention-to-treat cohort; *N* = 163) and the propensity score matched control cohort (*N* = 163)

Parameter	Study Regimen	Baseline	Follow-up	Difference (95% CI)	Treatment effect (95% CI)
Pre HD SBP [mmHg]	aligned	145.17+/−20.05	143.95+/−20.57	−1.22 (−5.65 to 3.2)	1.19 (−1.07 to 3.44)
	unaligned	145.12+/−19.33	142.7+/−19.7	−2.41 (−6.66 to 1.84)	
Post HD SBP [mmHg]	aligned	135.1+/−17.72	134.36+/−17.68	−0.74 (−4.6 to 3.12)	1.21 (−0.86 to 3.28)
	unaligned	137.08+/−18.12	135.1+/−18.3	−1.95 (−5.92 to 2.02)	
Pre HD DBP [mmHg]	aligned	74.12+/−12.25	73.59+/−12.16	−0.53 (−3.19 to 2.13)	0.3 (−0.91 to 1.51)
	unaligned	75.62+/−11.47	74.8+/−11.1	−0.83 (−3.3 to 1.63)	
Post HD DBP [mmHg]	aligned	69+/−10.39	68.83+/−10.49	−0.17 (−2.45 to 2.1)	0.43 (−0.67 to 1.53)
	unaligned	71.39+/−10.21	70.8+/−10.1	−0.6 (−2.82 to 1.61)	
Pre HD PP [mmHg]	aligned	71.06+/−14.92	70.36+/−15.52	−0.69 (−4.01 to 2.62)	0.89 (−0.59 to 2.36)
	unaligned	69.5+/−14.11	67.9+/−14.9	−1.58 (−4.74 to 1.58)	
Post HD PP [mmHg]	aligned	66.1+/−12.89	65.53+/−13.09	−0.57 (−3.4 to 2.27)	0.78 (−0.59 to 2.15)
	unaligned	65.69+/−13.27	64.3+/−13.6	−1.35 (−4.27 to 1.58)	
Pre HD MAP [mmHg]	aligned	97.8+/−13.58	97.04+/−13.64	−0.76 (−3.73 to 2.21)	0.6 (−0.88 to 2.08)
	unaligned	98.79+/−12.96	97.4+/−12.7	−1.36 (−4.16 to 1.44)	
Post HD MAP [mmHg]	aligned	91.03+/−11.82	90.67+/−11.81	−0.36 (−2.94 to 2.21)	0.69 (−0.66 to 2.04)
	unaligned	93.29+/−11.83	92.2+/−11.8	−1.05 (−3.63 to 1.52)	
Intra HD SBP change [mmHg]	aligned	−10.07+/−13.7	−9.59+/−13.34	0.48 (−2.46 to 3.43)	0.02 (−2.12 to 2.16)
	unaligned	−8.04+/−13.94	−7.6+/−13.5	0.46 (−2.53 to 3.45)	
Intra HD DBP change [mmHg]	aligned	−5.12+/−6.81	−4.76+/−6.47	0.36 (−1.09 to 1.8)	0.13 (−0.9 to 1.15)
	unaligned	−4.24+/−6.59	−4+/−6.5	0.23 (−1.2 to 1.66)

**Table 8:** Treatment effect of dialysate to serum sodium alignment on volume-related and hemodynamic parameters in those patients part to this quality initiative (as-treated cohort; *N* = 137) and the propensity score matched control cohort (*N* = 137)

Parameter	Study Regimen	Baseline	Follow-up	Difference (95% CI)	Treatment effect (95% CI)
Pre HD SBP [mmHg]	aligned	143.42+/−21.27	143.06+/−21.2	−0.36 (−5.41 to 4.69)	1.68 (−0.87 to 4.23)
	unaligned	146.94+/−20.88	144.9+/−22	−2.04 (−7.15 to 3.07)	
Post HD SBP [mmHg]	aligned	132.8+/−16.83	133.36+/−17.68	0.56 (−3.55 to 4.67)	1.43 (−0.86 to 3.72)
	unaligned	137.57+/−19.27	136.7+/−19.7	−0.87 (−5.5 to 3.76)	
Pre HD DBP [mmHg]	aligned	73.23+/−13.03	72.99+/−12.65	−0.24 (−3.3 to 2.81)	0.43 (−0.94 to 1.8)
	unaligned	75.9+/−12.16	75.2+/−12.1	−0.67 (−3.56 to 2.21)	
Post HD DBP [mmHg]	aligned	68.14+/−9.59	68.23+/−10.13	0.09 (−2.26 to 2.44)	0.34 (−0.91 to 1.58)
	unaligned	70.97+/−10.62	70.7+/−10.3	−0.25 (−2.74 to 2.24)	
Pre HD PP [mmHg]	aligned	70.19+/−15.3	70.07+/−15.6	−0.12 (−3.79 to 3.56)	1.25 (−0.4 to 2.9)
	unaligned	71.05+/−15.24	69.7+/−15.9	−1.37 (−5.07 to 2.34)	
Post HD PP [mmHg]	aligned	64.66+/−13.4	65.13+/−13.7	0.47 (−2.75 to 3.69)	1.09 (−0.38 to 2.57)
	unaligned	66.59+/−13.91	66+/−14.4	−0.62 (−4 to 2.75)	
Pre HD MAP [mmHg]	aligned	96.63+/−14.56	96.35+/−14.23	−0.28 (−3.71 to 3.14)	0.85 (−0.83 to 2.52)
	unaligned	99.58+/−13.86	98.5+/−14.3	−1.13 (−4.47 to 2.22)	
Post HD MAP [mmHg]	aligned	89.7+/−10.76	89.94+/−11.44	0.25 (−2.4 to 2.89)	0.7 (−0.82 to 2.22)
	unaligned	93.17+/−12.49	92.7+/−12.4	−0.45 (−3.41 to 2.51)	
Intra HD SBP change [mmHg]	aligned	−10.61+/−13.72	−9.7+/−14.67	0.92 (−2.46 to 4.3)	−0.25 (−2.65 to 2.14)
	unaligned	−9.38+/−14.23	−8.2+/−14.7	1.17 (−2.27 to 4.61)	
Intra HD DBP change [mmHg]	aligned	−5.09+/−6.8	−4.76+/−7.3	0.33 (−1.34 to 2.01)	−0.09 (−1.24 to 1.05)
	unaligned	−4.92+/−6.98	−4.5+/−7	0.43 (−1.23 to 2.09)

### Secondary outcomes

There were no significant differences between the groups at baseline for all-cause and fluid overload-related hospitalization rates in the ITT analysis. In the AT analysis fluid overload-related hospitalization rates differed significantly at BL (*P* = 0.02), while all-cause hospitalization rates did not. Analysis of the changes from BL to the follow-up period showed no significant treatment effect on all-cause hospitalization rates in both ITT and AT analysis, but a significant treatment effect on fluid overload-related hospitalization rates in the ITT analysis that was not found in the AT analysis (Table 9).

**Table 9:** Hospitalization rates in all studied patient cohorts

		Aligned			Not Aligned			Aligned versus not aligned
		Baseline	Follow-up	Change	Baseline	Follow-up	Change	Difference of changes from baseline to follow-up
**Intention to Treat**								
All-Cause Hospitalization	Total Admission Length [days]	3.93+/−8.7	3.18+/−6.22	−0.75 (−2.4 to 0.89)	6.12+/−13.35	5.92+/−10.25	−0.2 (−2.79 to 2.4)	−0.56 (−3.28 to 2.16)
	Hospitalization Rate [n/patient year]	1.92+/−3.15	1.66+/−2.62	−0.26 (−0.89 to 0.37)	2.3+/−3.5	2.58+/−3.68	0.28 (−0.5 to 1.06)	−0.54 (−1.38 to 0.3)
Fluid Overload-related Hospitalization	Total Admission Length [days]	0.36+/−2.19	0.05+/−0.44	−0.31 (−0.65 to 0.04)	0.32+/−1.32	0.87+/−3.07	0.55 (0.03 to 1.06)	−0.85 (−1.47 to −0.23)
	Hospitalization Rate [n/patient year]	0.17+/−0.78	0.04+/−0.34	−0.13 (−0.26 to 0)	0.19+/−0.73	0.45+/−1.44	0.26 (0.01 to 0.51)	−0.39 (−0.67 to −0.12)
**As Treated**								
All-Cause Hospitalization	Total Admission Length [days]	3.18+/−8.02	3.21+/−6.74	0.03 (−1.73 to 1.79)	5.23+/−12.92	5.56+/−9.31	0.34 (−2.34 to 3.02)	−0.31 (−3.03 to 2.42)
	Hospitalization Rate [n/patient year]	1.62+/−3.11	1.78+/−2.75	0.16 (−0.54 to 0.85)	1.87+/−3.03	2.67+/−4	0.8 (−0.04 to 1.64)	−0.64 (−1.51 to 0.22)
Fluid Overload-related Hospitalization	Total Admission Length [days]	0.33+/−2.34	0.2+/−1.07	−0.12 (−0.56 to 0.31)	0.83+/−4.07	1.31+/−4.85	0.48 (−0.58 to 1.55)	−0.61 (−1.75 to 0.53)
	Hospitalization Rate [n/patient year]	0.11+/−0.68	0.18+/−0.96	0.07 (−0.13 to 0.26)	0.4+/−1.21	0.6+/−1.84	0.2 (−0.17 to 0.57)	−0.13 (−0.53 to 0.27)

The Cox regression for repeated events showed non-significantly lower risk of hospitalization in those being aligned in the ITT analysis [0.84 (95% CI 0.65 to 1.09); *P* = 0.18] and AT [0.87 (95% CI 0.64 to 1.19); *P* = 0.38]. This lower risk remained consistent during the follow-up period in both groups, however, when comparing the risk between BL and follow-up within the groups no significant differences were found for both ITT and AT analyses. A borderline significant protective effect of alignment in the FOH in the aligned group [HR after alignment with BL as the referent 0.22 (95% CI 0.05 to 1.03); *P* = 0.05] is contrasting a non-significant but rather adverse effect of alignment in the AT analysis [HR after alignment with BL as the referent 1.60 (95% CI 0.52 to 4.89); *P* = 0.41].

### Tertiary outcomes

In the primary ITT and the secondary AT analysis there were no significant treatment effects on the neutrophil to lymphocyte ratio as an inflammatory marker.

A small lowering treatment effect on saline administration for those subject to alignment in the AT analysis [− 31.1 (95% CI -61.2 to − 0.9) mL is mainly attributable to an increase in saline administration in the unaligned patient group (472 ± 137 to 499 ± 169 mL).

### Subset analyses

In the subset analysis of those with a BL GNa^+^ greater than 1 mEq/L the treatment effect in both cohorts on IDWG was slightly more pronounced (Additional file 2: Table S1 and Table S1). The same held true for a subset analysis for those with pre HD SBP greater than 150 mmHg (Additional file 2: Table S2a and Table S2b**)**. Other results did not differ materially .

## Discussion

### Statement of principal findings

This retrospective propensity score-matched cohort study analyzing data of a quality improvement project is, to the best of our knowledge, the largest analysis of its kind. The current data confirms preliminary results from the same study cohort [11] and is, despite its retrospective design, suggestive about some of the effects likely to be seen in prospective observations.

Our results confirm earlier observations on the effects of sodium loading on serum osmolality resulting in increased thirst [7]. We found a consistently lowering treatment effect from sodium alignment on IDWG, confirming that a reduction in sodium loading, by only slightly altering the DNa^+^ prescription, may result in a decreased urge to drink (Table 3). The effects on other outcomes including hospitalizations remain somewhat unclear at this point and only allow speculations on what effects may be seen in analyses on a larger scale.

### Comparison to other studies

The reduction in IDWG observed in aligned patients were to some extent comparable to the results of a single-blinded cross-over study conducted by de Paula and colleagues [9] who reported a significant reduction in IDWG of 0.62 L on average, which is a larger effect than observed in our data [Table 3; − 0.1 (95% CI -0.2 to 0)]. However, this may be due to the study design as DePaula may have had tighter controls on SNa^+^ and SNa^+^ changes. In addition, they monitored the dialysate delivery with the aid of dialysate conductivity monitoring, which was not done in this project, which was more focused on insights in terms of efficiency. However in line with work by Edwards et al. who analyzed the clinical effects of systematic calibration of dialysate sodium delivery by the dialysis machine [18], one may speculate on how the lack of calibration and the possible resulting inaccuracy of delivery may have reduced the actual reduction of the diffusive sodium flux and thus the effect size of the intervention on IDWG.

DePaula and colleagues and Murisasco et al. reported improvement in SBP management in patients with higher SBP without increased occurrence of intradialytic morbid events. It is known that the intradialytic SNa^+^ concentration affects SBP which explains the main concern of compromised hemodynamic stability when lowering DNa^+^. We did not see effects on blood pressures in our data which dissipates this concern. However, given the effects on IDWG it may be speculated that in larger sample sizes and longer observation periods positive lowering effects on blood pressure such an effect may be seen. In addition, it is of note that alignment was implemented without causing an increased need for saline administration and thus likely does not cause an increased occurrence of intradialytic morbid events. While prospective studies confirming these specific findings are still needed, it seems to be at variance to the hypothesized positive effects of prescribing higher DNa^+^ in subjects with low SNa^+^ to promote hemodynamic stability as proposed by Hecking et al. [5, 19]. Since hemodynamic stability is a major concern of healthcare workers and physicians in the context of DNa^+^ to SNa^+^ alignment we believe these data are of great importance. The argument that sodium loading is more effective in preservation of hemodynamic stability as compared to an aligned dialysate sodium concentration with lower IDWGs and ultrafiltration needs, may not only be doubted in the light of our analyses but also in the light of earlier data [20]. While it is true that high dialysate sodium (exceeding SNa^+^ by 7%) preserves more plasma volume and the nadir correlates positively with the dialysate sodium used (with comparable blood pressures at the end of the treatment), van Stone and colleagues have shown that only a neutral GNa^+^ allows fluid removal exclusively from the ECV (where excess fluid accumulated during the interdialytic period) without substantial alteration of aldosterone levels [of note substantially decreasing during HD with high DNa^+^ [20]]. The authors of this report also concluded that low DNa^+^ results in an increased occurrence of intradialytic symptomatic hypotension while aligned and high DNa^+^ did not [20].

Given the relationship between fluid intake and sodium loading we also hypothesized relationships between the GNa^+^ and fluid overload (and the consequent increased hospitalization risk). Unfortunately, we did not have objective markers of fluid overload assessed in our subjects. Furthermore, the postdialysis target weight prescription was at the physician’s discretion and not part of this quality improvement. Given the lack of treatment effect one can assume a lack of effect on volume management, however, on a large scale the effects may very well be found. Lacson et al. reported that a nationwide reduction of DNa^+^, which was embraced by medical directors on a voluntary basis in their clinics, resulted in 592 dialysis clinics to lower DNa^+^, which resulted in a reduction of the median DNa^+^ by 3 mEq/L, in a significant reduction of IDWG and fluid-overload related hospitalizations [12]. Our data showed no effects on all-cause and fluid overload related hospitalization risk in the follow-up period between aligned and unaligned patients. However, in line with the findings of Lacson and colleagues one may speculate on the improvements by alignment in conjunction with targeted volume management and dietary sodium restriction. In addition, it may be noted that our data, along with those reported by Lacson and colleagues, is at variance to the report by the DOPPS study which claimed a rather counterintuitive decrease in risk to first all-cause and fluid overload-related hospitalization with the use of a higher DNa^+^.

Bedushi et al. found significant effects on inflammatory markers (i.e. Interleukin-6 and Tumor-Necrosis-Factor α) conferred by a reduction of DNa^+^. As a surrogate of these markers we decided to analyze the neutrophil-lymphocyte ratio but did not find any treatment effect in the studied cohorts.

### Study limitations

Despite the current analysis representing the largest sample studied to this date (until data from the SoLID trial [21] becomes available), several limitations need to be noted. For example, documentation errors may be possible from retrospective data extractions from medical records; but it is of note that the medical records were reviewed manually, which reduces the risk. In addition, there was a lack of information on the nature of intradialytic morbid events is noted and a detailed report of occurrences was not available, but we believe that the quantification of saline administration, as the major method to counteract intradialytic morbid events, was sufficiently conclusive. Another limitation was the lack of data on antihypertensive medication that were prescribed at the physician’s discretion however no changes to the preexisting prescriptions were expected. No assumptions on generalizability can be made at this point as only patients from one state (CT) were included in the analyses. Finally, as the prescription of the post-HD target weight remained at the treating physician’s discretion, the treatment effect on the pre- and post-HD weights should be interpreted cautiously.

## Conclusions

While intradialytic sodium loading is only one of the aspects beside to dietary and water-intake control and adequate dialysis prescription that affect and ameliorates the management of chronic fluid overload in dialysis patients, results from this DNa^+^ to SNa^+^ alignment program, which is to the best of the authors’ knowledge the largest to date, suggest favorable effects of such an intervention on several clinical factors. Well-matched control patients using propensity score-matching based on relevant parameters were studied to minimize confounding effects. It is important to note that the favorable effects found came without an increase in saline administration (used as a surrogate of the occurrence of intradialytic morbid events). The lack of effect on all-cause and fluid overload-related hospitalizations and the small effects on other parameters may be explained by a low baseline DNa^+^, limiting the interventional change of gradient. Additional confirmatory research in larger prospective studies is warranted.

## Additional files

Additional file 1: **Figure S1.** Quantile-quantile plot visually comparing the probability distribution of the quantiles of the study cohort and the propensity score-matched control cohort in the intention-to-treat analysis. **Figure S2.** Quantile-quantile plot visually comparing the probability distribution of the quantiles of the study cohort and the propensity score-matched control cohort in the as-treated analysis. (PPTX 470 kb)

Additional file 2: **Table S1 a).** Subset Analysis in those with a GNa^+^ > 1 mEq/L: Treatment effect of dialysate to serum sodium alignment in in those patients part to this quality initiative (intention-to-treat cohort; *N* = 66) and the propensity score matched control cohort (*N* = 64). **Table S1b).** Subset Analysis in those with a GNa^+^ > 1 mEq/L: Treatment effect of dialysate to serum sodium alignment in in those patients part to this quality initiative (as-treated cohort; *N* = 52) and the propensity score matched control cohort (N = 64). **Table S2a).** Subset Analysis in those with a pre HD SBP > 150 mmHg: Treatment effect of dialysate to serum sodium alignment in in those patients part to this quality initiative (intention-to-treat cohort; *N* = 50) and the propensity score matched control cohort (*N* = 78). **Table S2b.** *Subset Analysis in those with a pre HD SBP > 150 mmHg:* Treatment effect of dialysate to serum sodium alignment in in those patients part to this quality initiative (as-treated cohort; *N* = 72) and the propensity score matched control cohort (*N* = 73). (DOCX 25 kb)

## Abbreviation

AT: As Treated
BL: Baseline
BP: Blood Pressure
DNa^+^: Dialysate Sodium Concentration
DOPPS: Dialysis Outcomes and Practice Patterns Study
eKt/V: equilibrated Kt/V
GNa^+^: Dialysate to Serum Sodium Gradient
HD: Hemodialysis
HR: Hazard Ratio
ICD: International Classification of Diseases
IDWG: Interdialytic Weight Gain
ITT: Intention to Treat
MAP: Mean Arterial Pressure
NJ: New Jersey
SBP: Systolic Blood Pressure
SNa^+^: Serum Sodium Concentration
SNa^+^_estimated_: Estimated Serum Sodium Concentration
USA: United States of America
