# Supplementary figures and images for: Effects of dialysate to serum sodium (Na+) alignment in chronic hemodialysis (HD) patients: retrospective cohort study from a quality improvement project
Source: BMC Nephrol. 2018 Apr 2;19:75. doi: 10.1186/s12882-018-0870-0 (PMC5879548; doi:10.1186/s12882-018-0870-0)

## Slide 1
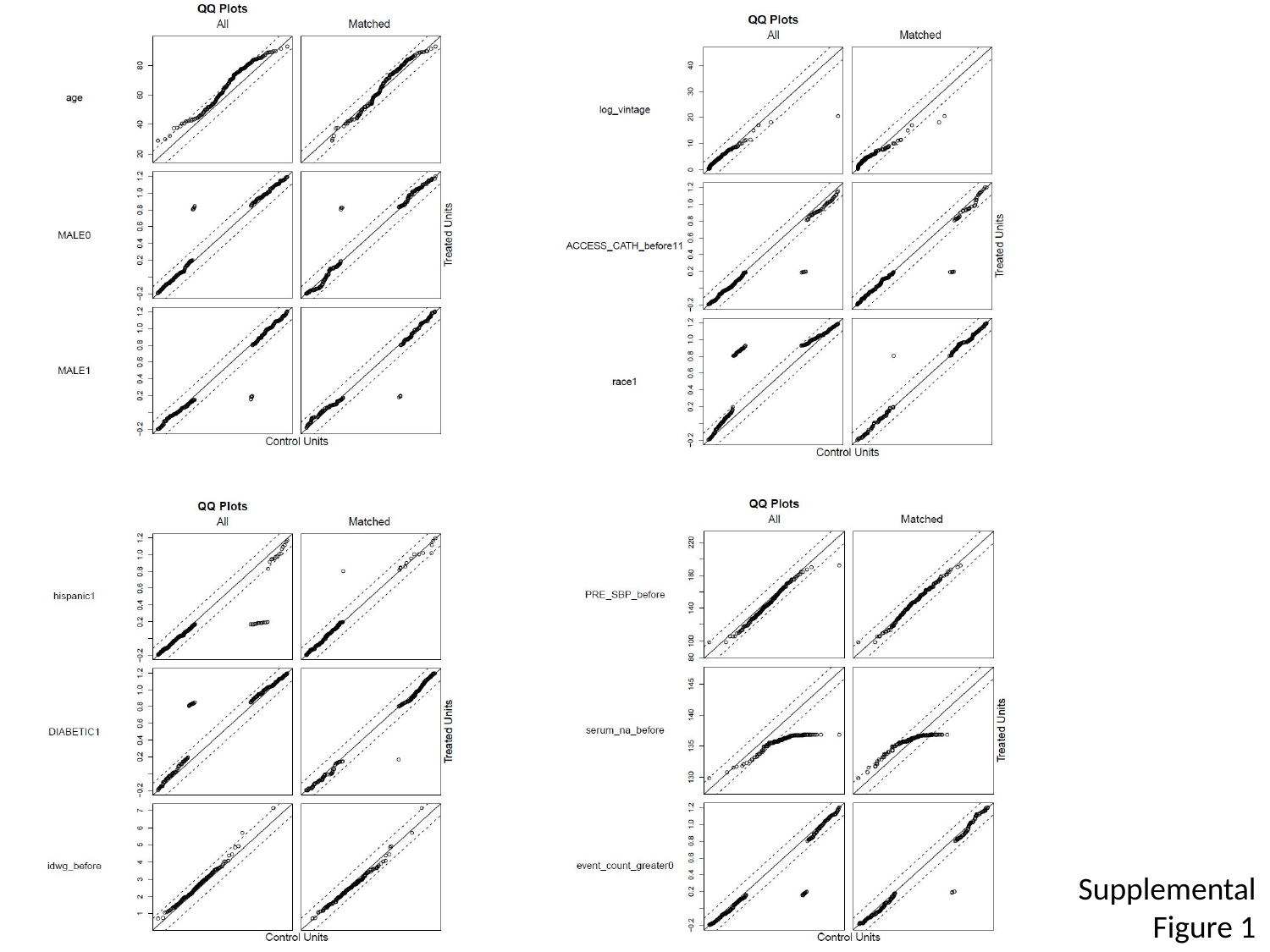

Supplemental
Figure 1

## Slide 2
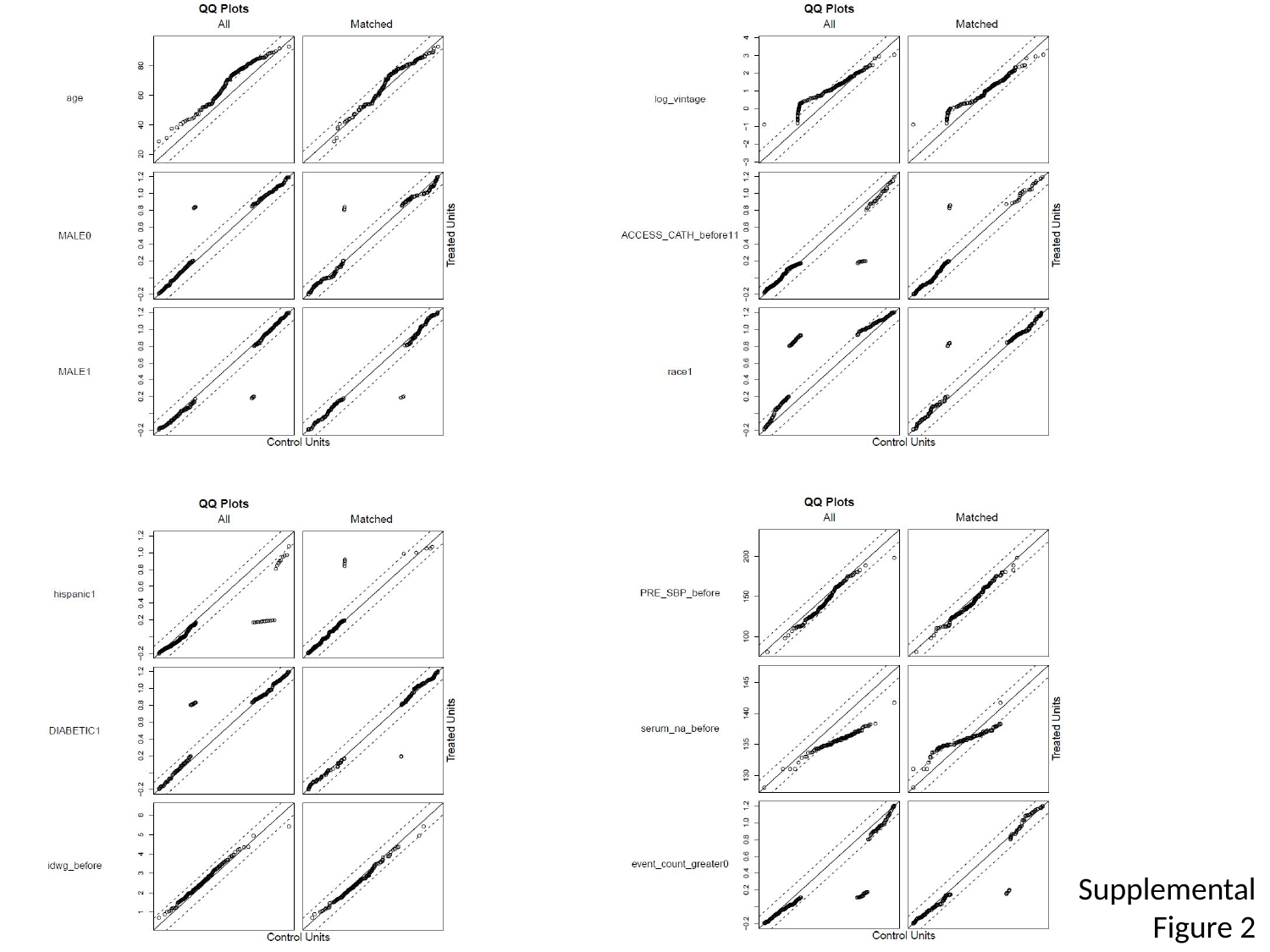

Supplemental
Figure 2

Supplement: Supplementary file 1 — Figure S1. Quantile-quantile plot visually comparing the probability distribution of the quantiles of the study cohort and the propensity score-matched control cohort in the intention-to-treat analysis. Figure S2. Quantile-quantile plot visually comparing the probability distribution of the quantiles of the study cohort and the propensity score-matched control cohort in the as-treated analysis. (PPTX 470 kb) [file 12882_2018_870_MOESM1_ESM.pptx]
