# Supplementary material for: Effects of dialysate to serum sodium (Na+) alignment in chronic hemodialysis (HD) patients: retrospective cohort study from a quality improvement project
Source: BMC Nephrol. 2018 Apr 2;19:75. doi: 10.1186/s12882-018-0870-0 (PMC5879548; doi:10.1186/s12882-018-0870-0)
Supplement: Supplementary file 2 — Table S1 a). Subset Analysis in those with a GNa+ > 1 mEq/L: Treatment effect of dialysate to serum sodium alignment in in those patients part to this quality initiative (intention-to-treat cohort; N = 66) and the propensity score matched control cohort (N = 64). Table S1b). Subset Analysis in those with a GNa+ > 1 mEq/L: Treatment effect of dialysate to serum sodium alignment in in those patients part to this quality initiative (as-treated cohort; N = 52) and the propensity score matched control cohort (N = 64). Table S2a). Subset Analysis in those with a pre HD SBP > 150 mmHg: Treatment effect of dialysate to serum sodium alignment in in those patients part to this quality initiative (intention-to-treat cohort; N = 50) and the propensity score matched control cohort (N = 78). Table S2b. Subset Analysis in those with a pre HD SBP > 150 mmHg: Treatment effect of dialysate to serum sodium alignment in in those patients part to this quality initiative (as-treated cohort; N = 72) and the propensity score matched control cohort (N = 73). (DOCX 25 kb) [file 12882_2018_870_MOESM2_ESM.docx]

**Supplemental Table 1 a):** Subset Analysis in those with a GNa>1mEq/L: Treatment effect of dialysate to serum sodium alignment in in those patients part to this quality initiative (intention-to-treat cohort; N=66) and the propensity score matched control cohort (N=64).

| Parameter | Study Regimen | Baseline | Follow-up | Difference (95% CI) | Treatment effect (95% CI) |
| --- | --- | --- | --- | --- | --- |
| IDWG [L] | aligned | 2.5+/-1.01 | 2.42+/-0.94 | -0.08 (-0.44 to 0.27) | -0.14 (-0.34 to 0.06) |
|  | unaligned | 2.57+/-0.81 | 2.6+/-0.9 | 0.06 (-0.19 to 0.31) |  |
| UFV [L] | aligned | 2.38+/-0.81 | 2.29+/-0.8 | -0.09 (-0.38 to 0.21) | -0.12 (-0.31 to 0.07) |
|  | unaligned | 2.49+/-0.83 | 2.5+/-0.9 | 0.03 (-0.21 to 0.27) |  |
| Pre HD wt [kg] | aligned | 82.24+/-26.5 | 81.38+/-26.05 | -0.87 (-10.53 to 8.8) | -0.23 (-1.63 to 1.17) |
|  | unaligned | 77.67+/-21.16 | 77+/-20 | -0.64 (-6.49 to 5.22) |  |
| Post HD wt [kg] | aligned | 79.87+/-26 | 78.91+/-24.98 | -0.96 (-10.34 to 8.42) | -0.25 (-1.65 to 1.15) |
|  | unaligned | 75.22+/-20.78 | 74.5+/-19.5 | -0.71 (-6.45 to 5.03) |  |
| Pre HD SBP [mmHg] | aligned | 147.75+/-18.87 | 145.45+/-19.13 | -2.3 (-9.29 to 4.68) | 0.19 (-3.47 to 3.86) |
|  | unaligned | 149.86+/-17.45 | 147.4+/-19.7 | -2.5 (-7.79 to 2.8) |  |
| Post HD SBP [mmHg] | aligned | 137.39+/-17.2 | 137.84+/-18.03 | 0.45 (-6.04 to 6.93) | 2.52 (-0.88 to 5.92) |
|  | unaligned | 142.21+/-16.45 | 140.1+/-16.9 | -2.07 (-6.82 to 2.67) |  |
| Pre HD DBP [mmHg] | aligned | 74.22+/-11.15 | 73.23+/-11 | -0.99 (-5.06 to 3.09) | 0.48 (-1.57 to 2.52) |
|  | unaligned | 78.14+/-10.39 | 76.7+/-10 | -1.46 (-4.37 to 1.45) |  |
| Post HD DBP [mmHg] | aligned | 69.13+/-8.83 | 69.74+/-9.8 | 0.61 (-2.82 to 4.04) | 1.91 (0.21 to 3.61) |
|  | unaligned | 74.2+/-9.32 | 72.9+/-9.1 | -1.3 (-3.92 to 1.32) |  |
| Pre HD PP [mmHg] | aligned | 73.53+/-14.42 | 72.22+/-15.26 | -1.32 (-6.78 to 4.14) | -0.28 (-2.74 to 2.17) |
|  | unaligned | 71.73+/-14.8 | 70.7+/-15.8 | -1.03 (-5.4 to 3.33) |  |
| Post HD PP [mmHg] | aligned | 68.26+/-13.01 | 68.1+/-13.26 | -0.16 (-4.99 to 4.67) | 0.61 (-1.71 to 2.94) |
|  | unaligned | 68.01+/-13.56 | 67.2+/-13.7 | -0.77 (-4.66 to 3.11) |  |
| Pre HD MAP [mmHg] | aligned | 98.73+/-12.46 | 97.3+/-12.29 | -1.42 (-5.98 to 3.13) | 0.38 (-2.05 to 2.82) |
|  | unaligned | 102.05+/-11.17 | 100.2+/-11.9 | -1.81 (-5.09 to 1.47) |  |
| Post HD MAP [mmHg] | aligned | 91.88+/-10.63 | 92.44+/-11.54 | 0.55 (-3.53 to 4.64) | 2.11 (-0.02 to 4.25) |
|  | unaligned | 96.87+/-10.35 | 95.3+/-10.4 | -1.56 (-4.51 to 1.4) |  |
| Intra HD SBP change [mmHg] | aligned | -10.36+/-14.09 | -7.61+/-15.11 | 2.75 (-2.62 to 8.13) | 2.33 (-1.6 to 6.25) |
|  | unaligned | -7.65+/-15.19 | -7.2+/-15.2 | 0.42 (-3.9 to 4.74) |  |
| Intra HD DBP change [mmHg] | aligned | -5.09+/-7.3 | -3.49+/-7.57 | 1.59 (-1.14 to 4.33) | 1.43 (-0.37 to 3.24) |
|  | unaligned | -3.93+/-7.35 | -3.8+/-6.8 | 0.16 (-1.86 to 2.18) |  |

**Supplemental Table 1b):** Subset Analysis in those with a GNa>1mEq/L: Treatment effect of dialysate to serum sodium alignment in in those patients part to this quality initiative (as-treated cohort; N=52) and the propensity score matched control cohort (N=64).

| Parameter | Study Regimen | Baseline | Follow-up | Difference (95% CI) | Treatment effect (95% CI) |
| --- | --- | --- | --- | --- | --- |
| IDWG [L] | aligned | 2.59+/-0.71 | 2.34+/-0.65 | -0.25 (-0.48 to -0.03) | -0.29 (-0.47 to -0.11) |
|  | unaligned | 2.69+/-0.84 | 2.7+/-1 | 0.04 (-0.26 to 0.33) |  |
| UFV [L] | aligned | 2.51+/-0.73 | 2.28+/-0.64 | -0.23 (-0.45 to 0) | -0.25 (-0.41 to -0.09) |
|  | unaligned | 2.6+/-0.89 | 2.6+/-0.9 | 0.02 (-0.27 to 0.31) |  |
| Pre HD wt [kg] | aligned | 80.11+/-17.75 | 79.35+/-16.56 | -0.75 (-6.41 to 4.9) | -0.03 (-1.2 to 1.13) |
|  | unaligned | 73.57+/-20.05 | 72.8+/-19 | -0.72 (-7.11 to 5.67) |  |
| Post HD wt [kg] | aligned | 77.62+/-17.41 | 77.09+/-16.36 | -0.53 (-6.1 to 5.03) | 0.24 (-0.9 to 1.38) |
|  | unaligned | 71.02+/-19.69 | 70.2+/-18.6 | -0.77 (-7.04 to 5.5) |  |
| Pre HD SBP [mmHg] | aligned | 142.36+/-21.22 | 142.63+/-20.79 | 0.27 (-6.65 to 7.19) | 2.82 (-1.03 to 6.68) |
|  | unaligned | 151.86+/-20.05 | 149.3+/-21.7 | -2.55 (-9.38 to 4.28) |  |
| Post HD SBP [mmHg] | aligned | 131.92+/-16.35 | 133.03+/-17.18 | 1.11 (-4.42 to 6.64) | 3.11 (-0.15 to 6.37) |
|  | unaligned | 141.67+/-18.76 | 139.7+/-18.3 | -2 (-8.07 to 4.06) |  |
| Pre HD DBP [mmHg] | aligned | 72.05+/-11.28 | 72.25+/-11.23 | 0.2 (-3.5 to 3.91) | 1.6 (-0.5 to 3.69) |
|  | unaligned | 78.81+/-11.36 | 77.4+/-11 | -1.39 (-5.06 to 2.27) |  |
| Post HD DBP [mmHg] | aligned | 67.03+/-8.06 | 67.57+/-8.52 | 0.54 (-2.19 to 3.28) | 1.62 (-0.22 to 3.46) |
|  | unaligned | 72.99+/-10.46 | 71.9+/-9.5 | -1.08 (-4.35 to 2.19) |  |
| Pre HD PP [mmHg] | aligned | 70.31+/-16.45 | 70.38+/-15.94 | 0.07 (-5.27 to 5.4) | 1.23 (-1.22 to 3.68) |
|  | unaligned | 73.06+/-16.42 | 71.9+/-17.1 | -1.16 (-6.65 to 4.33) |  |
| Post HD PP [mmHg] | aligned | 64.89+/-13.64 | 65.45+/-14.26 | 0.57 (-4.03 to 5.16) | 1.49 (-0.59 to 3.57) |
|  | unaligned | 68.68+/-14.37 | 67.8+/-14.6 | -0.92 (-5.66 to 3.82) |  |
| Pre HD MAP [mmHg] | aligned | 95.49+/-13.22 | 95.71+/-13.1 | 0.23 (-4.11 to 4.56) | 2.01 (-0.55 to 4.56) |
|  | unaligned | 103.16+/-12.65 | 101.4+/-13.1 | -1.78 (-6 to 2.44) |  |
| Post HD MAP [mmHg] | aligned | 88.66+/-9.55 | 89.39+/-10.08 | 0.73 (-2.5 to 3.97) | 2.12 (-0.08 to 4.32) |
|  | unaligned | 95.88+/-12.02 | 94.5+/-11.2 | -1.39 (-5.18 to 2.41) |  |
| Intra HD SBP change [mmHg] | aligned | -10.44+/-14.85 | -9.61+/-15.49 | 0.84 (-4.16 to 5.84) | 0.29 (-3.38 to 3.95) |
|  | unaligned | -10.19+/-15.17 | -9.6+/-16.6 | 0.55 (-4.65 to 5.75) |  |
| Intra HD DBP change [mmHg] | aligned | -5.02+/-6.98 | -4.68+/-7.59 | 0.34 (-2.06 to 2.74) | 0.02 (-1.62 to 1.67) |
|  | unaligned | -5.82+/-7.45 | -5.5+/-7.7 | 0.31 (-2.17 to 2.8) |  |

**Supplemental Table 2a):** Subset Analysis in those with a pre HD SBP>150 mmHg: Treatment effect of dialysate to serum sodium alignment in in those patients part to this quality initiative (intention-to-treat cohort; N=50) and the propensity score matched control cohort (N=78).

| Parameter | Study Regimen | Baseline | Follow-up | Difference (95% CI) | Treatment effect (95% CI) |
| --- | --- | --- | --- | --- | --- |
| IDWG [L] | aligned | 2.67+/-0.86 | 2.5+/-0.76 | -0.17 (-0.44 to 0.11) | -0.27 (-0.49 to -0.05) |
|  | unaligned | 2.58+/-0.93 | 2.7+/-1 | 0.1 (-0.24 to 0.45) |  |
| UFV [L] | aligned | 2.6+/-0.83 | 2.44+/-0.75 | -0.17 (-0.43 to 0.1) | -0.2 (-0.38 to -0.01) |
|  | unaligned | 2.56+/-0.91 | 2.6+/-0.9 | 0.03 (-0.3 to 0.36) |  |
| Pre HD wt [kg] | aligned | 80.54+/-19 | 79.34+/-18.64 | -1.2 (-7.53 to 5.14) | -0.29 (-1.73 to 1.15) |
|  | unaligned | 76.92+/-20.17 | 76+/-19.6 | -0.91 (-7.92 to 6.11) |  |
| Post HD wt [kg] | aligned | 77.93+/-18.63 | 76.88+/-18.35 | -1.06 (-7.28 to 5.17) | -0.08 (-1.5 to 1.34) |
|  | unaligned | 74.44+/-19.85 | 73.5+/-19.2 | -0.98 (-7.86 to 5.91) |  |
| Pre HD SBP [mmHg] | aligned | 164.27+/-10.63 | 159.84+/-13.04 | -4.43 (-8.44 to -0.42) | 0.33 (-3.97 to 4.63) |
|  | unaligned | 163.36+/-11.11 | 158.6+/-17.7 | -4.76 (-9.99 to 0.47) |  |
| Post HD SBP [mmHg] | aligned | 147.99+/-15.41 | 146.08+/-14.23 | -1.9 (-6.9 to 3.09) | 1.18 (-2.85 to 5.2) |
|  | unaligned | 149.2+/-15.93 | 146.1+/-17.4 | -3.08 (-8.96 to 2.79) |  |
| Pre HD DBP [mmHg] | aligned | 81.42+/-11.63 | 79.5+/-11.44 | -1.92 (-5.8 to 1.97) | 0.35 (-1.88 to 2.58) |
|  | unaligned | 82.67+/-9.74 | 80.4+/-10.3 | -2.27 (-5.8 to 1.26) |  |
| Post HD DBP [mmHg] | aligned | 73.9+/-9.89 | 73.26+/-10.31 | -0.64 (-4.04 to 2.76) | 0.53 (-1.5 to 2.56) |
|  | unaligned | 76.17+/-10.33 | 75+/-10.2 | -1.17 (-4.79 to 2.45) |  |
| Pre HD PP [mmHg] | aligned | 82.85+/-10.72 | 80.34+/-13.1 | -2.51 (-6.54 to 1.52) | -0.02 (-2.73 to 2.69) |
|  | unaligned | 80.69+/-12.4 | 78.2+/-15.2 | -2.49 (-7.39 to 2.41) |  |
| Post HD PP [mmHg] | aligned | 74.09+/-12.97 | 72.82+/-11.96 | -1.27 (-5.47 to 2.94) | 0.65 (-2 to 3.3) |
|  | unaligned | 73.03+/-12.97 | 71.1+/-13.8 | -1.91 (-6.63 to 2.81) |  |
| Pre HD MAP [mmHg] | aligned | 109.04+/-10.12 | 106.28+/-10.29 | -2.76 (-6.19 to 0.68) | 0.35 (-2.45 to 3.15) |
|  | unaligned | 109.56+/-8.38 | 106.5+/-11.1 | -3.1 (-6.58 to 0.37) |  |
| Post HD MAP [mmHg] | aligned | 98.59+/-10.34 | 97.53+/-10.33 | -1.06 (-4.54 to 2.42) | 0.75 (-1.82 to 3.32) |
|  | unaligned | 100.51+/-10.87 | 98.7+/-11.3 | -1.81 (-5.72 to 2.1) |  |
| Intra HD SBP change [mmHg] | aligned | -16.29+/-15.82 | -13.76+/-15.8 | 2.53 (-2.8 to 7.85) | 0.85 (-3.06 to 4.76) |
|  | unaligned | -14.16+/-16.46 | -12.5+/-15.7 | 1.68 (-4 to 7.36) |  |
| Intra HD DBP change [mmHg] | aligned | -7.52+/-8.08 | -6.24+/-7.83 | 1.28 (-1.4 to 3.96) | 0.18 (-1.69 to 2.04) |
|  | unaligned | -6.5+/-7.53 | -5.4+/-7.3 | 1.1 (-1.51 to 3.71) |  |

**Supplemental Table 2b):** Subset Analysis in those with a pre HD SBP>150 mmHg: Treatment effect of dialysate to serum sodium alignment in in those patients part to this quality initiative (as-treated cohort; N=72) and the propensity score matched control cohort (N=73).

| Parameter | Study Regimen | Baseline | Follow-up | Difference (95% CI) | Treatment effect (95% CI) |
| --- | --- | --- | --- | --- | --- |
| IDWG [L] | aligned | 2.8+/-0.94 | 2.58+/-0.79 | -0.22 (-0.56 to 0.11) | -0.26 (-0.48 to -0.04) |
|  | unaligned | 2.81+/-0.94 | 2.8+/-1 | 0.03 (-0.3 to 0.37) |  |
| UFV [L] | aligned | 2.75+/-0.94 | 2.52+/-0.77 | -0.23 (-0.56 to 0.11) | -0.24 (-0.43 to -0.04) |
|  | unaligned | 2.77+/-0.93 | 2.8+/-0.9 | 0.01 (-0.31 to 0.33) |  |
| Pre HD wt [kg] | aligned | 84.31+/-22.47 | 82.87+/-22.08 | -1.45 (-10.11 to 7.22) | -1.29 (-2.43 to -0.14) |
|  | unaligned | 73.97+/-16.9 | 73.8+/-17.2 | -0.16 (-6.11 to 5.8) |  |
| Post HD wt [kg] | aligned | 81.55+/-21.87 | 80.35+/-21.74 | -1.2 (-9.68 to 7.28) | -1.01 (-2.13 to 0.1) |
|  | unaligned | 71.24+/-16.53 | 71.1+/-16.7 | -0.19 (-6 to 5.62) |  |
| Pre HD SBP [mmHg] | aligned | 165.65+/-10.45 | 163+/-12.44 | -2.65 (-7.12 to 1.82) | 2.05 (-1.86 to 5.97) |
|  | unaligned | 164.78+/-11.39 | 160.1+/-17.1 | -4.7 (-9.79 to 0.38) |  |
| Post HD SBP [mmHg] | aligned | 146.21+/-13.42 | 146.49+/-14.81 | 0.28 (-5.22 to 5.78) | 2.11 (-1.87 to 6.08) |
|  | unaligned | 149.88+/-16.01 | 148.1+/-16.8 | -1.83 (-7.58 to 3.92) |  |
| Pre HD DBP [mmHg] | aligned | 82.97+/-12.92 | 81.95+/-12.78 | -1.01 (-6.01 to 3.99) | 0.8 (-1.41 to 3.02) |
|  | unaligned | 83.3+/-10.91 | 81.5+/-11.7 | -1.82 (-5.78 to 2.14) |  |
| Post HD DBP [mmHg] | aligned | 73.97+/-9.98 | 74.08+/-10.78 | 0.11 (-3.93 to 4.15) | 0.87 (-1.25 to 2.98) |
|  | unaligned | 76.19+/-11.03 | 75.4+/-10.9 | -0.75 (-4.58 to 3.08) |  |
| Pre HD PP [mmHg] | aligned | 82.68+/-12.28 | 81.04+/-13.03 | -1.64 (-6.56 to 3.29) | 1.25 (-1.28 to 3.78) |
|  | unaligned | 81.48+/-11.72 | 78.6+/-13.3 | -2.89 (-7.26 to 1.49) |  |
| Post HD PP [mmHg] | aligned | 72.24+/-13.28 | 72.41+/-13.12 | 0.16 (-4.97 to 5.3) | 1.24 (-1.22 to 3.7) |
|  | unaligned | 73.7+/-11.55 | 72.6+/-12.6 | -1.08 (-5.31 to 3.16) |  |
| Pre HD MAP [mmHg] | aligned | 110.53+/-10.68 | 108.97+/-11.08 | -1.56 (-5.79 to 2.67) | 1.22 (-1.42 to 3.86) |
|  | unaligned | 110.46+/-9.59 | 107.7+/-12.2 | -2.78 (-6.63 to 1.07) |  |
| Post HD MAP [mmHg] | aligned | 98.05+/-9.34 | 98.22+/-10.6 | 0.17 (-3.72 to 4.05) | 1.28 (-1.35 to 3.91) |
|  | unaligned | 100.75+/-11.7 | 99.6+/-11.7 | -1.11 (-5.21 to 2.99) |  |
| Intra HD SBP change [mmHg] | aligned | -19.43+/-14.69 | -16.5+/-17.49 | 2.93 (-3.36 to 9.21) | 0.05 (-4.31 to 4.42) |
|  | unaligned | -14.9+/-15.09 | -12+/-16 | 2.87 (-2.57 to 8.32) |  |
| Intra HD DBP change [mmHg] | aligned | -9+/-7.58 | -7.87+/-8.88 | 1.13 (-2.09 to 4.34) | 0.06 (-1.96 to 2.09) |
|  | unaligned | -7.12+/-7.14 | -6.1+/-6.9 | 1.07 (-1.39 to 3.52) |  |
